# Acceptability of Components for a Mandatory Quality Improvement Framework: A Survey Among Swiss General Practitioners

**DOI:** 10.1177/11786329251346828

**Published:** 2025-06-21

**Authors:** David Wirth, Oliver Senn, Jakob M. Burgstaller, Sima Djalali, Leander Muheim, Adrian Rohrbasser, Joel Lehmann, Stefan Markun

**Affiliations:** 1 Institute of Primary Care, University and University Hospital Zurich, Zurich, Switzerland; 2 Zollikerberg Hospital, Clinic for Internal Medicine/Interdisciplinary Emergency Ward and SGAIM Quality Commission, Zollikerberg, Switzerland; 3 mediX zürich AG, Zürich, Switzerland; 4 Medbase Wil Friedtal, Wil, Switzerland; 5 EQUAM Stiftung, Bern, Switzerland

**Keywords:** quality improvement, primary health care; general practitioners, physician, health care quality, access, and evaluation, surveys and questionnaires, quality assurance, health care, health policy, Switzerland

## Abstract

**Background:**

In Switzerland, recently introduced legislation requires the implementation of a framework for mandatory quality improvement at the level of individual general practitioners (GPs) and includes the introduction of quality indicators (QIs) amongst other components. The GP-sided acceptance of potential components of such a framework is important to its success.

**Objectives:**

To identify components of a potential framework for mandatory quality improvement that are most likely to be accepted by Swiss GPs.

**Design:**

Cross-sectional web-based survey conducted among employed and self-employed Swiss GPs in 2024.

**Methods:**

The survey was distributed to 1103 Swiss GPs via their physician networks. The survey inquired the acceptability of 62 possible components of a mandatory framework for quality improvement. Components were categorized as “acceptable” if they were rated as “acceptable” or “very acceptable” by more than 50% of participants, in contrast to those rated as “neutral” or “not acceptable.”

**Results:**

A total of 244 GPs participated (participation rate 22.1%, 53.0% male, 51.2% <50 years old, 50.8% employed). The majority of participants rated 31 of the proposed 62 components as acceptable. Among these were QIs pertaining to structures and processes of care (rated as acceptable by 58.3%-83.4%) and sharing QI achievement data with peers from different group practices and physician networks (53.9%-92.2%). A majority of participants accepted physician networks, medical associations, and academic institutions as entities that could establish QIs and manage QI data (acceptance 62.1%-88.8%).

**Conclusions:**

Swiss GPs appear to accept QIs that reflect structures and processes of care established by physician networks, medical associations or academic institutions, exclusively shared among their peers.

## Introduction

In most high-income countries, primary healthcare is provided by general practitioners (GPs) and maintaining efficiency and quality of service in this setting remains a constant challenge. High-quality healthcare should be effective, safe and person-centered,^1^ but maintaining such quality requires significant investment in human resources, infrastructure, and work organization.^2,3^ National health policies and legislation are cornerstones for quality improvement, as they provide the regularory framework for progress in quality initiatives.^4^ In Switzerland, a recent revision of the Health Insurance Act (Article 58a) created a legal obligation for all health care providers to continuously measure and disclose quality data and to implement and disclose continuous quality improvement activities.^5^ At the time of writing, the exact implementation of this legislation and its impact on GPs are unclear and depend on the outcome of ongoing negotiations between medical associations and healthcare insurance associations and subsequent approval by the Swiss federal government.

There are several key components that need to be considered for the implementation of a national framework for mandatory quality improvement, and the views of key stakeholders need to be taken into account since organizational resistance from their side is amongst the most important obstacles.^6^ The key stakeholders in the Swiss context are the health insurance companies and the physicians both represented by their respective associations in the negotiations on the implementation of Article 58a. In this respect, GPs are the most notable key stakeholders, as they represent up the majority of the physician workforce in Switzerland and will therefore inevitably bear the brunt of the new regulation’s administrative burden. A framework that is acceptable to GPs is therefore important, as increasing the administrative burden will lead to workforce attrition in a profession that is already in short supply.^7-13^ It is therefore important to consider the views of GPs on components of a framework for mandatory quality improvement as regulated by the Health Insurance Act. In particular, a better understanding is needed of GPs’ willingness to accept specific components of quality measurement and reporting, such as quality indicators (QIs), and their acceptance of the different stakeholders involved in the process of developing QIs and collecting and sharing QI achievement data.

In addition, in Switzerland, as in other healthcare systems across western Europe^14,15^ and North America,^16^ GPs’ preferences are shifting from self-employment toward employed work. A number of reasons have been proposed for this trend toward employment, including increasing economic constraints in the general practice sector as well as the changing demographics of the physician workforce itself, which is seeking more flexible and family-friendly working conditions and work structures that reduce administrative burdens and allow more focus on patient care. Consequently, GPs may have different preferences regarding mandatory quality improvement depending on their employment state, especially because of the potential for increased administrative burden, which could be highly relevant for policy implementation.^17^ Furthermore, as employed GPs are often in larger group practices with a homogeneous shared infrastructure, large-scale quality improvement may be more efficient to implement, but acceptance among these GPs remains key to success.

Given the foreseeable need to implement recent legislative changes regarding mandatory quality improvement in different Swiss general practice settings, we sought to identify possible framework components that are most acceptable from the perspective of GPs. Specifically, the aims of this study were primarily to identify potential framework components of mandatory quality improvement which are considered “acceptable” by a majority of GPs. Secondarily, the study aimed to test the hypothesis that acceptance of framework components is associated with employment status (self-employed vs employed).

## Methods

### Study Design

A web survey (cross-sectional study) was conducted among Swiss GPs. The survey methods were designed according to the CHERRIES instrument - —a 30-item checklist designed to guide and improve reporting and methodology of web-based survey research. The CHERRIES checklist focusses on specific common concerns for online surveys such as multiple potential methods for calculating a response rates and offers guidance to improve transparency and reproducibility of research.^18^ Participants were recruited through physician networks, and invited to participate in the survey which was available up on the a secure, web-based REDCap (Research Electronic Data Capture) platform hosted by our institution. A separate survey collector was created for each physician network to allow our research department to certify that the survey was actually distributed within each physician network. We approached the mediX network and the Medbase group. The mediX network is the largest physician network in German-speaking Switzerland, consisting of predominantly self-employed GPs and organized into 10 sub-networks in different geographical areas. The Medbase group is a major healthcare provider in Switzerland, offering general practice services and having the single largest number of employed Swiss GPs. In addition, to collect data from smaller GP organizational units, we approached GPs who were not part of either the mediX network or the Medbase group, using a convenience sample of GPs affiliated with our research organization through scientific collaborations. These GPs were members of a diverse collection of smaller physician lead networks with heterogeneous management systems, varying in administrative processes, resource allocation strategies, and degrees of centralization in decision-making, with a generally higher emphasis on individual autonomy and practitioner-dependent quality measures.

The inclusion criterion for respondents was to be working as a GP and was operationalized at the level of the invitation emails. Respondents were excluded if they abandoned the questionnaire without answering any of the QI questions. This study being a survey among physicians not delivering interventions or treatments and not containing any individual’s health-related data was outside the scope of the Swiss Federal Act on Research involving Human Beings (as defined in Art. 2) and does not meet the criteria for research that requires ethics committee approval or GCP-compliance, which are meant for clinical investigations involving patients rather than surveys among physicians.^19^ Participation in this survey was voluntary. Informed consent was obtained from respondents prior to the start of the survey through written information in the invitation e-mail sent to GP networks forwarded to GPs. Specifically, participants were informed that responses would be anonymized, summarized, and published. As an incentive to participate, 3 vouchers worth CHF 300 each were raffled among participants.

### Instruments and Procedures

The questionnaire was written in German and was developed on the basis of existing literature on quality activities in Switzerland,^12^ and in collaboration with a panel of experts involved in quality improvement in the Society of General Internal Medicine (SND), in physician networks (LM and AR) or in a Swiss healthcare quality accreditation organization (JL). The comprehensibility of the questionnaire was improved by piloting it and conducting focused interviews with 12 GPs from the target group selected for convenience.

The questionnaire (Supplemental Material) consisted of 86 items, 24 of which asked about GP demographics (age, sex), type of practice (single- vs group practices, employment status), years of experience, number of colleagues in the practice, previous experience of quality management (range of options given), preferred format for publication of QI data (Static reporting eg, PDF-file, interactive reporting eg, dashboard), and perceived need for continuous quality improvement in general practice (five-point Likert scale). The remaining 62 items asked GPs to indicate their level of acceptance for different potential components of a framework for mandatory quality improvement on a five-point Likert scale (ranging from “completely unacceptable” to “completely acceptable,” with a central option for “neutral/cannot answer”). Specifically, the items were grouped into sections relating to types of QIs (structural, process, and outcome indicators), entities with whom data on QI achievement could be shared (physician network, insurers, federal government, public etc.), entities involved in the establishment of QIs as well as the collection and management of related data (physician network, insurers, federal government, academic institutions etc.), and possible incentives relating to quality improvement. In order to explain the terminology, the items contained examples where necessary. The questionnaire was developed between October and December 2023 and piloted in January 2024. The questionnaire was eventually distributed to the participants between February and June 2024 via the respective central organizational units (the mediX sub-networks, the Medbase group or our institution, respectively) by e-mail using an individual survey collector for each organizational unit linking to the REDCap survey platform hosted by our institution.^20,21^ After an initial invitation to complete the survey, a reminder to participate was sent after 1 or 2 weeks. There was no time limit to complete the survey once opened, and respondents could review and change their answers until they were finished using the “back” button. No method was implemented to account for eventual duplicate entries. The survey was closed on 30 June 2024. The data was retrieved from the REDcap server by SM who deleted the optional variable containing participants’ email addresses (for GPs who opted to participate in the raffle) for anonymization before proceeding with the statistical analysis.

### Objectives and Statistical Analysis

We report overall GP characteristics and general views on quality improvement, and tabulate results grouped by employment status (self-employed vs employed) using counts and proportions (n and %) or means and standard deviations (SD). We present ratings unchanged using data visualizations. To meet our primary objective (to identify potential framework components of mandatory quality improvement which are considered “acceptable” by a majority of GPs), we dichotomized Likert scales contrasting “acceptable” with other ratings (“rather acceptable” and “completely acceptable” ratings combined as “acceptable” and other ratings including the neutral as “neutral or unacceptable”) and report contrasts of self-employed versus employed GPs in tabular form (Supplemental Material).

To meet our secondary objective (to test whether the dichotomized response rates were associated with employment), we used chi-squared tests and logistic regression models. The outcome variable was the dichotomized acceptance rating, and the determinant was employment status (self-employed vs employed). To account for potential differences in demographic characteristics impacting the model results, the models were adjusted for GP sex, years of practice experience, and previous participation in quality improvement initiatives.

To address multiple testing, *P*-values from the model outputs were adjusted using the Bonferroni method. A corrected threshold was used to determine statistical significance, where *P*-values were compared against α/n, with α = .05, and n being the number of tests. An unadjusted threshold of α = .05 was applied to results from chi-squared tests.

We used the R statistical program (R version 4.4.1 and RStudio 2024.09.0 Build 375) for all analyses, using the *tidyverse, tableone*, and *likert* libraries.^22^

## Results

### Sample Characteristics and General Views

We invited a total of 1103 GPs to complete the questionnaire, 255 responded (recruitment rate 23.1%) by starting the questionnaire and 223 participated by answering at least 1 item asking about QIs and were thus included in the study (participation rate 20.2%). Of the participating GPs, 201 completed the survey (completeness rate 90.1%). The included GPs, 50.8% reported working as employees, 53.0% were male, 51.2% < 50 years of age and 62.1% possessed at least 10 years of working experience as GPs. Of the participants, 43.4% agreed that continuous quality improvement is necessary in general practice, 33.6% disagreed and 23.0% were neutral. Regarding time allocated to quality improvement, 74.9% answered 1 hour per month should not be exceeded. Characteristics of the included participants are listed in Table 1. Notably, employed GPs were significantly younger (*P* = .029), more often female (*P* < .001), less often board certified general internists (*P* = .001) and less experienced in previous quality initiatives (*P* = .048).

**Table 1.** Participant Characteristics and General Views.

Characteristic	Level	Self-employed	Employed	*P*
N		121	102	
Age (%)	Age <40	14 (12.2)	21 (23.9)	.029
	Age 40-49	35 (30.4)	34 (38.6)	
	Age 50-59	41 (35.7)	20 (22.7)	
	Age ⩾60	25 (21.7)	13 (14.8)	
Sex (%)	Female	40 (34.8)	55 (63.2)	<.001
	Male	75 (65.2)	32 (36.8)	
Board certification in general internal medicine (%)	Yes	111 (89.5)	76 (63.3)	<.001
	No	13 (10.5)	44 (36.7)	
Other board certificate (%)	Yes	14 (11.3)	3 (2.5)	.014
	No	110 (88.7)	117 (97.5)	
Years of experience as GP (%)	0-10 y	38 (33.0)	39 (44.3)	.127
	10-20 y	37 (32.2)	29 (33.0)	
	>20 y	40 (34.8)	20 (22.7)	
Electronic medical record (%)	Yes	114 (99.1)	88 (100.0)	1
	No	1 (0.9)	0 (0.0)	
Practice type (%)	Single practice	25 (20.2)	0 (0.0)	<.001
	Group practice (employed)	0 (0.0)	120 (100.0)	
	Group practice (self-employed)	99 (79.8)	0 (0.0)	
Number of colleagues in same practice (mean (SD))		3.3 (3.4)	7.1 (5.5)	<.001
Agreement to necessity of continuous quality improvement in general practice (%)	Agree	46 (37.1)	56 (50.5)	.054
	Neutral or disagree	78 (62.9)	55 (49.5)	
Previous participation in quality improvement initiatives (%)	Yes	103 (83.1)	87 (72.5)	.067
	No	21 (16.9)	33 (27.5)	
Interactive report format (e.g. dashboard; %)	Acceptable	63 (52.1)	64 (63.4)	.119
	Neutral or unacceptable	58 (47.9)	37 (36.6)	
Static report format (e.g. PDF; %)	Acceptable	55 (45.5)	57 (56.4)	.135
	Neutral or unacceptable	66 (54.5)	44 (43.6)	
Maximum monthly time willing to allocate to quality development (%)	0 h	31 (27.0)	15 (17.0)	.019
	<1 h	63 (54.8)	43 (48.9)	
	1-2 h	19 (16.5)	23 (26.1)	
	Up to half a day	1 (0.9)	7 (8.0)	
	Up to 1 d	1 (0.9)	0 (0.0)	

### Different Types of QIs

**Figure 1.**
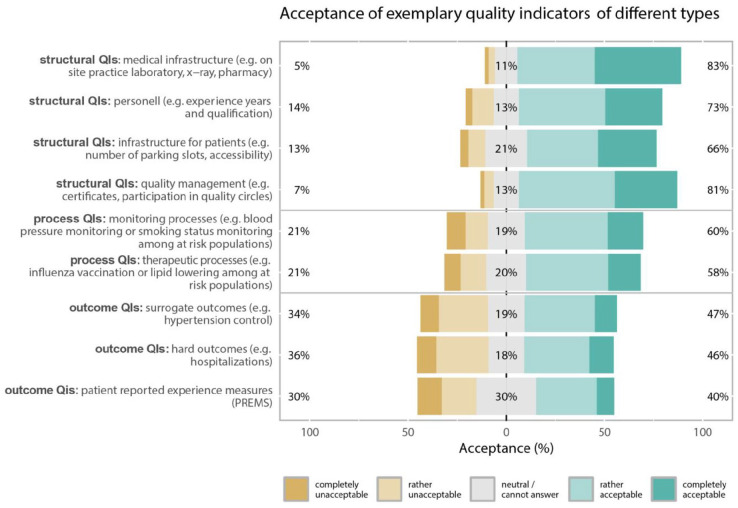
Likert plot for questions asking about acceptance of different types of quality indicators (QIs): participants’ acceptance ratings of the QI types are represented in the horizontal bars and the numbers in % (rounded) at the side and in the center summarize the response categories (left = % rather unacceptable or completely unacceptable, center = % neutral/cannot answer, right = % rather acceptable or fully acceptable).

Among different illustrative types of QIs, all structural indicators and process indicators were rated as acceptable by a majority of participants (proportions ranging from 58.3% to 83.4%, Figure 1). On the other hand, outcome indicators had a comparatively lower level of acceptance (proportions ranging from 39.7% to 47.1%). There was no statistically significant difference between employed and self-employed GPs with respect to acceptance of types of quality indicators (Supplemental Table 1).

### Sharing QI Achievement Data

**Figure 2.**
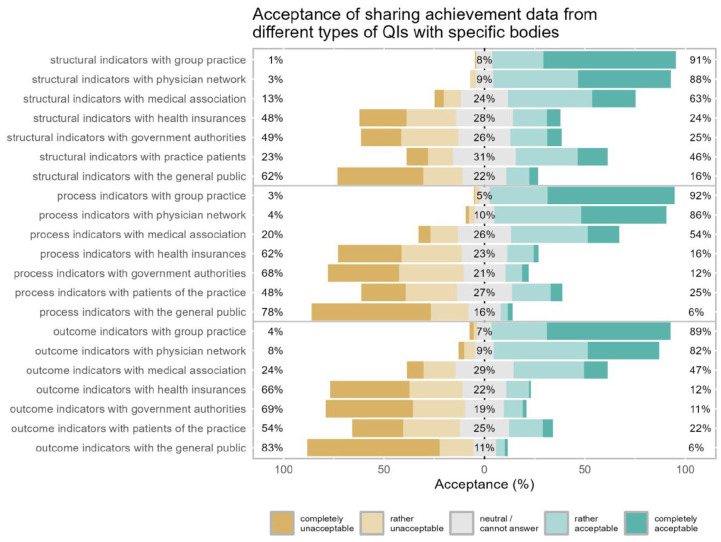
Acceptance of sharing achievement data from different types of QIs with specific bodies. Likert plot for questions asking about acceptance of sharing QI achievement data of different types of quality indicators (QIs) with different entities: participants’ acceptance ratings are represented in the horizontal bars and the numbers in % (rounded) at the side and in the center summarize the response categories (left = % rather unacceptable or completely unacceptable, center = % neutral/cannot answer, right = % rather acceptable or fully acceptable).

The majority of participants found it acceptable to share QI performance data with peers in the group practice or peers in the same physician network for all 3 types of QIs (acceptance ranged between 82.3% and 92.2%, Figure 2). Sharing QI achievement data with medical associations was acceptable for the majority only in the case of structural and process indicators (63.5% and 53.9%, respectively). Sharing achievement data of any other QI type with any other entity had substantially lower acceptance. And the acceptance of sharing with the general public was especially low (structural QI data 15.9%, process QI data 5.9% and outcome QI data 5.9%). Employed GPs showed significantly higher acceptance compared to self-employed GPs with regard to sharing achievement data of structural QIs with patients of the practice (54.9% vs 38.5%, *P* = .026). Moreover, employed GPs tended to be significantly more accepting toward sharing achievement of process indicators with healthcare insurance companies (27.0% vs 7.0%, *P* < .001) or government authorities (21.3% vs 4.3%, *P* < .001; Supplemental Table 2).

### Establishing QIs, Gathering, and Managing QI Data

**Figure 3.**
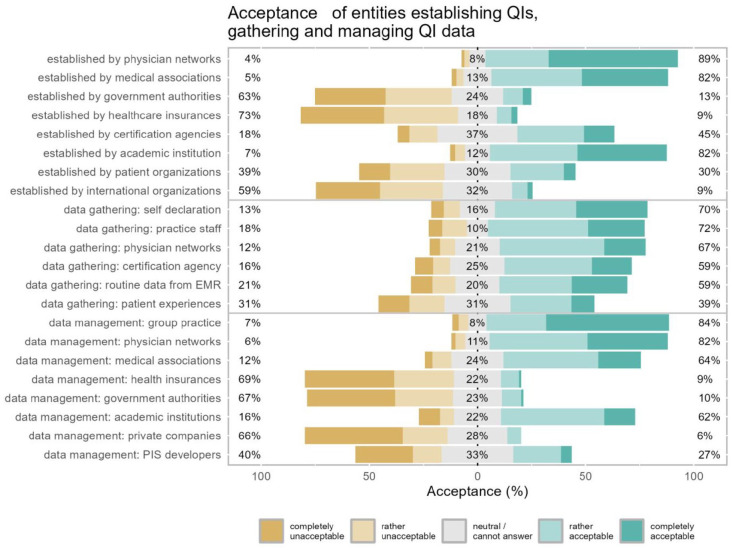
Acceptance of entities establishing QIs, gathering and managing QI data. Likert plot for questions asking about acceptance of entities establishing quality indicators (QIs), gathering and managing QI data: participants’ acceptance ratings are represented in the horizontal bars and the numbers in % (rounded) at the side and in the center summarize the response categories (left = % rather unacceptable or completely unacceptable, center = % neutral/cannot answer, right = % rather acceptable or fully acceptable). EMR: electronic medical records, PIS: practice information system.

A majority of participants rated physician networks, medical associations and academic institutions as acceptable entities to establish QIs (acceptance of 88.8%, 81.6% and 81.6%, respectively, Figure 3). Regarding the data source to measure QI achievement, all proposed options with the exception of patient experience measures were viewed as acceptable by more than 50% of participants. Organizational entities for management of QI data considered acceptable by the majority of participants were group practices, physician networks, medical associations, and academic institutions (84.2%, 82.3%, 63.5%, and 62.1% respectively). Employed GPs compared to self-employed GPs tended to be significantly more accepting toward QIs being established by healthcare insurances (16.7% vs 3.3%, *P* = .002) or government authorities (20.6% vs 6.6%, *P* = .004) or certification agencies (50.4% vs 36.4%, *P* = .008). Moreover, employed GPs tended to be significantly more accepting toward QI achievement data being managed by healthcare insurances (18.2% vs 2.6%, *P* < .001) or government authorities (19.3% vs 2.6%, *P* < .001; Supplemental Table 3).

### Incentives

**Figure 4.**
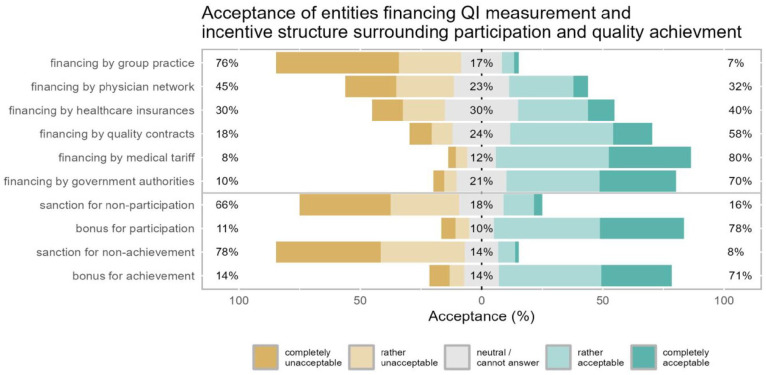
Acceptance of financing sources and incentives. Likert plot for questions asking about acceptance of financing sources and incentives for quality improvement: participants’ acceptance ratings are represented in the horizontal bars and the numbers in % (rounded) at the side and in the center summarize the response categories (left = % rather unacceptable or completely unacceptable, center = % neutral/cannot answer, right = % rather acceptable or fully acceptable).

With respect to incentives, a majority of participants deemed financing quality improvement by medical tariff adaptations, government authorities or quality contracts with healthcare insurance companies as acceptable (80.4%, 69.9%, and 58.4%, respectively, Figure 4). A bonus system incentivizing participation in quality improvement as well as achievement of quality goals was rated as acceptable by the majority of participants (78.2% and 71.3%). Self-employed and employed GPs did not differ significantly from each other (Supplemental Table 4).

### Association of Employment State With Acceptance of Framework Components

Multivariate logistic regression models revealed that 21 out of 62 framework components were more likely to be acceptable by employed GPs (Supplemental Table 5 and Supplemental Figure 1), however after adjustment for multiple testing, only 4 of these remained statistically significant: These were first and second: Sharing achievement rates from process QIs with healthcare insurances companies (OR 5.9, CI: 2.5-15.3, *P* = .008), or with government authorities (OR 7.4, CI: 2.7-24.4, *P* = .018), third: involvement of government authorities in QI data management (OR 9.9, CI: 3.0-45.3. *P* = .038), and fourth: involving healthcare insurances companies in the establishment of QIs (OR 7.8, CI: 2.6-29.8, *P* = .048).

## Discussion

In this study, we sought to identify the components of a framework for mandatory quality improvement that would be most acceptable for the majority of Swiss GPs and to explore the potential differences between employed and self-employed GPs in this regard. In terms of type of QI acceptance, we found that the majority of GPs participating in the study would accept QIs that reflect structures and processes of care, and that they would accept sharing their QI achievement data with peers from their group practices and physician networks. Furthermore, the majority of respondents would accept physician networks, medical associations and academic institutions as entities that establish QIs and manage QI data. As funding entities, the majority of respondents would accept adjustments to the medical tariff, government funding or funding through quality contracts with healthcare insurance companies. In addition, the majority of respondents would accept a financial bonus mechanism to incentivize participation in a quality improvement process and achievement of quality goals. Compared with self-employed GPs, employed GPs were more willing to accept involvement of healthcare insurances companies and government authorities in specific components of quality improvement.

In numerous international and European health care systems, including the USA,^23,24^ the United Kingdom,^25^ France,^26^ or the Netherlands,^27^ QI achievement monitoring at the GP-level has been implemented in a continuous, coordinated and publicly disclosed manner. Various efforts exist in Switzerland, but transparency, coordination, feedback, and relevance to the general practice setting are generally lacking.^28^ Specifically, at the time of writing, publicly available QI data exist in Switzerland, for example, in the form of the Swiss Health Care Atlas,^29^ annual reports published by healthcare insurance companies^30^ or by the government.^31^ However, these data typically are aggregated at a large geographic level or limited to hospitals, leaving the role of individual health care providers completely unclear. There are projects where GP-level data are reflected to GPs, but these are fragmented and lack external transparency.^32,33^ In contrast, in a recent pilot project, Swiss ambulatory care providers disclosed quality improvement activities comparable to structural QIs, but without disclosing actual QI achievement data, leaving it unclear how these activities were actually sustained.^12^

Regarding the type of QI and the publicity of reporting, even though intended by policy-makers, the study participants seem to clearly reject framework components that imply sharing of QI achievement data on a publicly available dashboard, as implemented in the Quality and Outcomes Framework in England.^25^ Reported reasons for rejecting such transparency, particularly with respect to outcome indicators, include the fact that they are prone to misinterpretations due to long delays between healthcare provision and measurably prevented adverse outcomes and lack of risk adjustment, which in the eyes of many clinicians severely impairs the potential to derive meaningful and concrete actions at the expense of additional costs.^34,35^ However, the majority of study participants would accept sharing QI achievement data among their peers in group practices, physician networks, or the medical society. This highlights the potential of motivating GPs to engage in quality improvement by introducing reputational incentives within their peer-groups, which was shown to be important for GPs.^36,37^ Interestingly, despite the legitimate criticism that QIs often measure processes of care rather than patient-relevant outcomes, study participants were actually more open to process indicators than outcome indicators. This suggests a higher barrier toward the possible implementation of outcome indicators which needs to be addressed. Interestingly, practice patients were the fourth most trusted group with whom study participants would accept sharing QI achievement data. Such transparency toward patients is intended to drive quality-mediated competition.^38,39^ Arguably, however, the relevance of such competition is small since patients tend to trade quality for convenience of practice locations or relationships to clinicians according to studies from the UK and the USA.^40,41^

For the majority of study participants, physician networks, medical associations, and academic institutions were acceptable entities for establishing QIs. This highlights the potential for these entities to coordinate and design quality improvement initiatives and communicate QI activities to Swiss general practice. Experience with existing QI frameworks based on claims data has shown, that a joint effort by healthcare insurance companies, physician networks, and academic institutions is a prerequisite for implementation,^42,43^ while medical associations have only published very limited activity.^44^ Interestingly, study participants were open to different institutions or methods of data collection, with the remarkable exception of patient experiences. This is surprising, as patient experiences are considered a central component of quality of care.^1^ Interestingly, a previous study has identified inaccuracies in GP understanding regarding subjective aspects of their patients’ health, which could also explain their lower acceptance of patient experience in a mandatory QI framework.^45^ Furthermore, we hypothesize that the low acceptance of patient experiences may be partially explained by GPs’ experiences with physician-rating websites and social media platforms which are often lacking transparency and quality standards themselves, since standardized patient reported experience measures (PREMS) are not yet broadly implemented in Switzerland.^46^ Among potential strategies to increase GPs acceptance of PREMS are perspective-taking exercises or interventions fostering empathy that can help physicians understand patients’ experiences more profoundly.^47-49^ However, to successfully increase GPs’ willingness to engage with PREMs the additional administrative burden imposed by measurement and data analysis should also be minimized for example by efficient integration into electronic medical records.^50^

Similar to data collection, the majority of study participants would accept their QI data being managed by a wide range of potential stakeholders, including themselves, physician networks, medical associations, and academic institutions. Interestingly, healthcare insurance companies, practice information system developers, and government agencies were not considered acceptable by a majority of study participants, which contrasts to the reality that these entities already possess and manage or have a legal right to obtain sufficient data to produce GP-level QIs.^5,42^

Perhaps not surprisingly, the majority of study participants would find sanctions for non-participation or non-achievement in quality improvement unacceptable, nor did a majority accept the financing of quality improvement with resources from their own network, or direct financing by healthcare insurance companies. On the other hand, the majority of study participants would accept a financing mechanism through quality contracts with healthcare insurance companies at the network level, which is a well-established model in Switzerland allowing network-level financial incentives and actionable practice change initiatives and is associated with improvements in quality of care.^51^ Study participants would also accept an individual reward system that provides incentives to participate in quality improvement initiatives and to achieve quality goals. In this regard, however, it should be noted that a recent study failed to demonstrate the effects of a direct pay-for-performance system that incentivized both process and outcome QIs for diabetes, hypertension, and hyperlipidemia in Switzerland.^52^ Even in the broader context, the evidence for meaningful effects of pay-for-performance incentives or financial incentives for GPs is inconsistent, pay-for-performance may even be counterproductive, and arguably the concept itself is based on flawed assumptions about the motivation of GPs.^34,53-57^ Especially concerning in this regard are the increasing time constraints and administrative workloads in general practice which not only compete directly with engagement in quality improvement but also indirectly by contributing to physician burnout.^58-60^ Finally, we found that the majority of study participants would accept quality improvement being financed by tariff adjustments or by the government. In the Swiss publicly financed health care system, both options would lead to increased public spending and public stakeholders would need to be convinced by such cost externalizations with according strategies. In this regard, the literature suggests that private-public partnerships or health impact bonds allowing governments to transfer implementation risks to private investors while only paying for successful outcomes could acceptable solutions.^61^

Compared to self-employed GPs, employed GPs in our study were more accepting of sharing QIs with a wide range of stakeholders outside of the practice including healthcare insurance companies, the government, and the general public. This observation is not surprising, given that employed GPs are already in a situation where data sharing and accountability with an overarching organizational structure are normalized. Notwithstanding these statistical differences, it is important to understand that the majorities for acceptance of almost all framework conditions were consistent in both groups of GPs, and especially a publicly available dashboard, as currently envisaged by some policy stakeholders, may find little acceptance among GPs regardless of employment modality. In contrast, a dashboard for sharing QIs exclusively within professional networks appears to acceptable.

A limitation of this study is the moderate response rate, which leaves the views of the majority of the GPs approached unclear. In this regard, we hypothesize that GPs without strong opinions on this issue may have tended to not participate, thus censoring their perspectives. In terms of external applicability, this means that the issue is likely to be perceived as less polarized than this study suggests, and thus resistance to implementing quality improvement is likely to be less of an issue. Furthermore, our sampling was mostly aimed at the largest GP networks from Switzerland. While these represent the most important groups in numbers and thus their responses carry a most important weight, representativeness for the total group of Swiss GPs cannot be claimed. The survey was conducted without a prior power calculation, which means that the sample size may not have been adequate to detect small or subtle effects. Consecutively, there is a potential for Type II errors (failure to detect true associations). Similarly, this study was conducted only in the German-speaking part of Switzerland, and the views of Italian-speaking and French-speaking Swiss GPs are not represented. Regardless, the majority opinions on the acceptability of quality improvement framework components were overwhelmingly clear in several instances.

## Conclusion

In conclusion, for a mandatory quality improvement framework to be successful and well-accepted by Swiss GPs, it should prioritize QIs focusing on structures and processes of care, emphasize peer-to-peer learning and data sharing within professional networks, and involve established medical and academic bodies in the development and management of QIs. Policymakers should also be mindful of the reservations surrounding public reporting and outcome indicators, and consider the differing perspectives between employed and self-employed GPs when designing and implementing such frameworks. Ultimately, incorporating these preferences is crucial for fostering a sense of ownership and engagement among Swiss GPs, which is essential for the effective implementation of mandatory quality improvement initiatives.

## Supplemental Material

sj-docx-1-his-10.1177_11786329251346828 – Supplemental material for Acceptability of Components for a Mandatory Quality Improvement Framework: A Survey Among Swiss General Practitioners Supplemental material, sj-docx-1-his-10.1177_11786329251346828 for Acceptability of Components for a Mandatory Quality Improvement Framework: A Survey Among Swiss General Practitioners by David Wirth, Oliver Senn, Jakob M. Burgstaller, Sima Djalali, Leander Muheim, Adrian Rohrbasser, Joel Lehmann and Stefan Markun in Health Services Insights

sj-docx-2-his-10.1177_11786329251346828 – Supplemental material for Acceptability of Components for a Mandatory Quality Improvement Framework: A Survey Among Swiss General Practitioners Supplemental material, sj-docx-2-his-10.1177_11786329251346828 for Acceptability of Components for a Mandatory Quality Improvement Framework: A Survey Among Swiss General Practitioners by David Wirth, Oliver Senn, Jakob M. Burgstaller, Sima Djalali, Leander Muheim, Adrian Rohrbasser, Joel Lehmann and Stefan Markun in Health Services Insights
