# Supplementary material for: Acceptability of Components for a Mandatory Quality Improvement Framework: A Survey Among Swiss General Practitioners
Source: Health Serv Insights. 2025 Jun 21;18:11786329251346828. doi: 10.1177/11786329251346828 (PMC12182608; doi:10.1177/11786329251346828)
Supplement: sj-docx-1-his-10.1177_11786329251346828 – Supplemental material for Acceptability of Components for a Mandatory Quality Improvement Framework: A Survey Among Swiss General Practitioners [file sj-docx-1-his-10.1177_11786329251346828.docx]

Rahmenbedingungen für ein Qualitätsdashboard in der Hausarztmedizin

**Herzlich willkommen zu unserer Befragung!**

Sehr geehrte Damen und Herren Liebe Kolleginnen und Kollegen

Die Hausarztmedizin sieht sich mit politischen und gesellschaftlichen Forderungen nach Qualitätstransparenz konfrontiert. Die Revision von Art. 58a KVG sieht dazu ein obligatorisches Qualitätsmonitoring auf Ebene der einzelnen Leistungserbringer vor (1). Die genaue Umsetzung von Artikel 58a ist zurzeit Gegenstand von Verhandlungen zwischen Ärzteschaft, Versicherern und Bund. Mit diesem Projekt möchten wir die Meinung der Ärzteschaft zu den Rahmenbedingungen einer konkreten Umsetzungsmöglichkeit einholen und damit die politische Diskussion voranbringen.

Eine mögliche Umsetzung ist ein Qualitäts-Dashboard (2), eine Benutzeroberfläche, auf der Kennzahlen zur Behandlungsqualität einzelner Leistungserbringer abrufbar sind. Qualitäts-Dashboards haben sich bereits in verschiedenen Bereichen der Medizin bewährt, insbesondere wenn die Ärzteschaft in den Gestaltungsprozess einbezogen wurde (3).

Zielsetzung: Das Ziel dieser Umfrage besteht darin, Sie als Hausärzt:innen bereits vor der Konzeption eines möglichen Qualitäts-Dashboards einzubeziehen. Insbesondere soll mit dieser Umfrage geklärt werden, welche Rahmenbedingungen aus Ihrer Sicht akzeptabel sind.

Hinweise zur Teilnahme an dieser Befragung: Wir legen grossen Wert auf den Schutz Ihrer Daten. Ihre Antworten in dieser Umfrage werden von Ihrer Email-Adresse und sonstigen identifizierenden Daten getrennt aufbewahrt und getrennt ausgewertet, sodass zu keiner Zeit Rückschlüsse auf ihre Person möglich sind. Die Umfrage dauert ca. 10 Minuten. Um Ihre wertvollen Zeitreserven zu schonen, haben wir die Fragen auf das wesentlichste reduziert. Unter den Teilnehmenden werden 3 Gutscheine im Wert von je 300 Franken für einen Anbieter Ihrer Wahl verlost.

Vielen Dank, dass Sie sich die Zeit nehmen, an dieser Befragung teilzunehmen. Mit Ihrer Teilnahme tragen Sie zur Gestaltung der Qualitätsmessung der medizinischen Versorgung in der Schweiz bei.

Falls Sie Fragen oder Bedenken haben, zögern Sie bitte nicht, uns zu kontaktieren. Ihre Meinung ist uns wichtig, und wir schätzen Ihre Unterstützung.

Mit freundlichen Grüssen

David Wirth dipl. med.

Institut für Hausarztmedizin Universitätsspital Zürich Pestalozzistr. 24

CH-8091 Zürich

E-Mail: [david.wirth@usz.ch](mailto:david.wirth@usz.ch)

1. Bundesamt für Gesundheit BAG, Erläuterungen Artikel 58a Absatz 2 KVG, (2022)
2. Qualitäts-Dashboards sind Websites, für die Anzeige von Kennzahlen der Behandlungsqualität. Die Information kann dabei aggregiert angezeigt oder mittels interaktiver Funktionen detailliert dargestellt werden. Ein

Qualitäts-Dashboard nutzt zur Darstellung der Analysen intuitive Visualisierungen und Tabellen. Ziel eines Qualitäts-Dashboards ist es letztlich, objektive, fundierte Vergleiche von Leistungserbringern zu ermöglichen und deren Qualitätsentwicklung zu fördern.

1. Becker B, Nagavally S, Wagner N, Walker R, Segon Y, Segon A. Creating a culture of quality: our experience with providing feedback to frontline hospitalists. BMJ Open Qual. 2021;10(1).

Klicken Sie bitte auf den Button "Nächste Seite >>", um die Befragung zu starten!

Systemgestaltung, Transparenz, Datenmanagement, Kosten & Aufwand Unserer Befragung befasst sich mit Systemgestaltung, Transparenz, Datenmanagement und Kosten eines potenziellen Qualitäts-Dashboard.

Praxis und Netzwerk

Frage 1: Welcher Art von Praxis gehören Sie an? Einzelpraxis Gruppenpraxis (angestellt)

Gruppenpraxis (selbständig / Mitinhaber:In) Andere


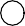

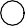

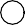

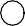


Bitte spezifizieren:

Frage 2: Wie viele weitere hausärztliche Kolleg:Innen arbeiten in Ihrer Praxis?

Frage 3: Mit welchem der Qualitätsmonitoring- und
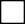
 EQUAM

Feedback-Systeme haben Sie bereits Erfahrungen gemacht
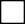
 FIRE-Feedback-Report (Mehrfachnennungen möglich)?
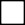
 ISO-Zertifizierung


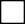
 EFQM


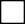
 Helsana Q-Benchmarking
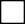
 Anderen


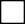
 Keinem

Welche(s)?

Frage 4: Wie fest stimmen Sie der folgenden Aussage Stimme überhaupt nicht zu für die Hausarztpraxis zu? Stimme eher nicht zu

neutral


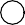

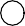

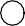

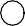

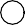

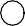


"Verbesserung der Versorgungsqualität durch Stimme eher zu

kontinuierliche Messung ist nötig." Stimme völlig zu

Kann ich nicht beantworten

**Frage 5: Um Qualität zu messen, muss entschieden werden, was genau gemessen werden soll. Wer soll mitentscheiden, was gemessen wird? Bewerten Sie die möglichen Entscheidungsträger nach ihrer Akzeptanz.**

völlig

inakzeptabel

eher

inakzeptabel

neutral eher

akzeptabel

voll und ganz

akzeptabel

kann ich nicht

beantworten

1.
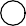

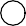

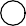

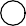

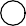

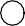
Ärztenetzwerke
2.
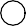

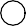

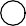

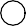

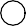

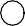
Fachgesellschaften
3.
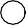

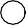

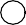

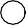

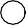

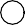

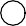

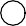

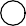

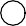

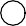

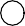
Kantons- oder dB)unKdraenskr egnikearsusnegn
4.
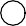

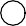

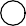

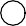

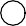

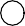
Zertifizierungsstelle (z.B. EQUAM)
5.
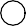

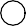

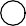

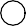

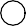

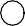
Akademische Institute für Hausarztmedizin
6.
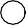

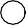

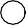

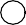

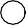

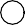
Patientenorganisationen
7.
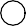

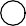

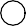

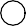

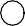
Internationale Organisationen
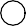


**Frage 6: Wie akzeptabel sind für Sie folgende mögliche BERICHTSFORMATE über Ihre Versorgungsqualität?**

völlig

inakzeptabel

eher

inakzeptabel

neutral eher

akzeptabel

voll und ganz

akzeptabel

kann ich nicht

beantworten

1.
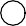

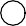

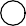

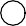

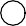

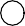
Statischer Bericht (z.B. PDF)
2.
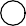

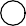

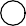

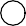

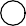

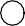
Interaktiver Bericht (z.B. elektronisches Dashboard)

| **Frage 7: Wie akzeptabel sind für Sie die folgenden möglichen FINANZIERUNGSSZENARIEN für**  **verbindliche Qualitätsmessung?** | | | | | | |
| --- | --- | --- | --- | --- | --- | --- |
|  | völlig inakzeptabel | eher inakzeptabel | neutral | eher akzeptabel | voll und ganz akzeptabel | kann ich nicht beantworten |
| a) Finanzierung durch Hausärzte/Gruppenpraxis (selbstzahlend) | 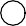 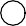 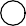 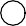 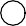 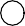  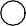 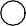 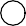 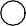 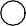 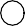  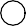 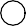 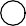 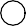 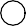 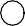  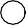 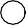 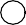 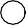 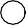 | | | | | |
| b) Finanzierung durch Ärztenetzwerke (selbstzahlend) |  |  |  |  |  |  |
| c) Finanzierung durch Krankenkassen (selbstzahlend) |  |  |  |  |  |  |
| d) Finanzierung durch Qualitätsverträge zwischen Hausärzten und Krankenkassen (finanziert durch qualitätsbedingte Einsparungen) |  |  |  |  |  |  |
| e) Finanzierung durch Tarifstruktur Tarmed/Tardoc, z.B. über generelle Tariferhöhung oder Einführung von spezifischen Qualitätsleistungen (öffentliche Kostenträgerschaft) |  |  |  |  |  |  |

f) Zusatzfinanzierung durch Bund oder Kanton (öffentliche Kostenträgerschaft)

**Fragensequenz 8-13: Akzeptanz von Struktur-, Prozess- und Outcomeindikatoren**

Die folgenden Fragen beziehen sich auf Struktur-, Prozess- und Outcome-Indikatoren. Diese Einteilung wird häufig für die Qualitätsmessung verwendet.

Strukturindikatoren (Frage 8 und 9): Strukturindikatoren geben Auskunft über Infrastruktur, Personal und übergeordnete Prozesse. Beispiele sind Angaben zur medizinischen Infrastruktur (Praxislabor, Röntgengeräte, Apotheke), zur personellen Infrastruktur (Anzahl, Erfahrung und Qualifikation der Ärzte und des medizinischen Personals) sowie zur patientenbezogenen Infrastruktur (Parkplätze, Barrierefreiheit). Darüber hinaus spiegelt sich das Qualitätsmanagement (Zertifizierungen, Teilnahme an Qualitätszirkeln) in den Strukturindikatoren wider.

Prozessindikatoren (Frage 10 und 11): Prozessindikatoren geben Einblick in durchgeführte Behandlungsprozesse; konkret in durchgeführte Überwachungsmassnahmen und Therapieprozesse. Beispiele sind Angaben zur Häufigkeit spezifischer Überwachungsprozesse (Blutdruckmessungen bei Patienten mit arterieller Hypertonie) und Therapieprozesse (Influenza-Impfungen bei COPD-Patienten, Statin-Verschreibungen bei KHK-Patienten). Diese Indikatoren helfen, die Umsetzung von Behandlungsrichtlinien zu bewerten.

Outcomeindikatoren (Frage 12 und 13): Outcomeindikatoren messen den Erfolg bei der Erreichung vordefinierter Behandlungsziele. Dabei wird zwischen Surrogat-Outcomes (z.B. Blutdruckziele bei Hypertonikern) und harten Outcomes (z.B. Hospitalisierungsraten bei COPD-Patienten) unterschieden. Darüber hinaus können Ergebnisse aus strukturierten Patientenbefragungen (PROMS) berücksichtigt werden. Diese Indikatoren geben Aufschluss über die Wirksamkeit medizinischer Interventionen.

| **Frage 8: STRUKTURINDIKATOREN bilden Infrastruktur, Personal und übergeordnete Prozesse**  **eines Leistungserbringers ab (siehe Beispiele). Welche Art von verbindlichen Strukturindikatoren halten Sie für akzeptabel?** | | | | | | |
| --- | --- | --- | --- | --- | --- | --- |
|  | völlig inakzeptabel | eher inakzeptabel | neutral | eher akzeptabel | voll und ganz akzeptabel | kann ich nicht beantworten |
| a) Angaben zur medizinischen Infrastruktur (z.B. Praxislabor, Röntgengeräte, Apotheke) |  | | | | | |
| b) Angaben zur personellen Infrastruktur (z.B. Anzahl, Erfahrungsjahre und Qualifikationen von Ärztinnen und Ärzten, MPKs, MPAs, Sekretariat) |  |  |  |  |  |  |
| c) Angaben zur Infrastruktur für Patientinnen und Patienten (z.B. Anzahl Parkplätze, Rollstuhlgängigkeit etc.) |  |  |  |  |  |  |
| d) Angaben zum Qualitätsmanagement (z.B. Zertifizierungen, Teilnahme an Qualitätszirkel) |  |  |  |  |  |  |

| **Frage 9: STRUKTURINDIKATOREN bilden Infrastruktur, Personal und übergeordnete Prozesse eines Leistungserbringers ab. Welche Stufe von Transparenz nach aussen halten Sie bei Strukturindikatoren für akzeptabel? "Meine Strukturindikatoren zu teilen finde ich akzeptabel**  **mit: ..."** | | | | | | |
| --- | --- | --- | --- | --- | --- | --- |
|  | völlig inakzeptabel | eher inakzeptabel | neutral | eher akzeptabel | voll und ganz akzeptabel | kann ich nicht beantworten |
| a) Ärzten und Ärztinnen in meiner Gruppenpraxis |  |  |  |  |  |  |
| b) meinem Ärztenetzwerk (Ärzte und Ärztinnen, Administration, Geschäftsführung, weitere Leistungserbringer) |  |  |  |  |  |  |
| c) allen Ärzten und Ärztinnen meines Dachverbandes |  |  |  |  |  |  |
| d) den Krankenkassen |  |  |  |  |  |  |
| e) der Kantons- oder Bundesregierung |  |  |  |  |  |  |
| f) den Patient:Innen der Praxis |  |  |  |  |  |  |
| g) der uneingeschränkten Allgemeinheit (z.B. frei zugängliche Daten im Internet) |  |  |  |  |  |  |

| **Frage 10: PROZESSINDIKATOREN bilden durchgeführte Behandlungsprozesse ab (siehe**  **Beispiele). Welche Art von verbindlichen Prozessindikatoren halten Sie für akzeptabel?** | | | | | | |
| --- | --- | --- | --- | --- | --- | --- |
|  | völlig inakzeptabel | eher inakzeptabel | neutral | eher akzeptabel | voll und ganz akzeptabel | kann ich nicht beantworten |
| a) Angaben zur Häufigkeit bestimmter ÜBERWACHUNGSPROZESSE bei  bestimmten Patientengruppen (z.B. Blutdruckmessungen bei Patienten mit arterieller Hypertonie, Anteil der  KHK-Patienten mit dokumentiertem Raucherstatus) |  |  |  |  |  |  |
| b) Angaben zur Häufigkeit bestimmter THERAPIEPROZESSE bei bestimmten Patientengruppen (z.B. Anteil der COPD-Patienten mit Influenza-Impfung; Anteil der KHK-Patienten, die Statine erhalten) |  |  |  |  |  |  |

| **Frage 11: PROZESSINDIKATOREN bilden durchgeführte Behandlungsprozesse ab. Welche Stufe**  **von Transparenz nach aussen halten Sie bei Prozessindikatoren für akzeptabel? "Meine Prozessindikatoren zu teilen finde ich akzeptabel mit: ..."** | | | | | | |
| --- | --- | --- | --- | --- | --- | --- |
|  | völlig inakzeptabel | eher inakzeptabel | neutral | eher akzeptabel | voll und ganz akzeptabel | kann ich nicht beantworten |
| a) Ärzten und Ärztinnen in meiner Gruppenpraxis |  |  |  |  |  |  |
| b) meinem Ärztenetzwerk |  |  |  |  |  |  |
| c) allen Ärzten und Ärztinnen meines Dachverbandes |  |  |  |  |  |  |
| d) den Krankenkassen |  |  |  |  |  |  |
| e) der Kantons- oder Bundesregierung |  |  |  |  |  |  |
| f) den Patient:Innen der Praxis |  |  |  |  |  |  |
| g) der uneingeschränkten Allgemeinheit (z.B. frei zugängliche Daten im Internet) |  |  |  |  |  |  |

| **Frage 12: OUTCOMEINDIKATOREN bilden den Erfolg im Erreichen vordefinierter**  **Behandlungsziele ab (siehe Beispiele). Welche Art von verpflichtenden Outcomeindikatoren halten Sie für akzeptabel?** | | | | | | |
| --- | --- | --- | --- | --- | --- | --- |
|  | völlig inakzeptabel | eher inakzeptabel | neutral | eher akzeptabel | voll und ganz akzeptabel | kann ich nicht beantworten |
| a) Häufigkeit der Erreichung bestimmter Surrogat-Outcomes bei bestimmten Patientengruppen (z.B. Anteil der Hypertoniker mit einem Blutdruck unter 140/90 mmHg) |  |  |  |  |  |  |
| b) Häufigkeit der Erreichung bestimmter harter Outcomes bei bestimmten Patientengruppen (z.B. Anteil der Patienten mit COPD welche wegen akuter Exazerbation hospitalisiert werden mussten) |  |  |  |  |  |  |
| c) Resultate von strukturierten Patientenbefragungen (z.B. PROMS) |  |  |  |  |  |  |

| **Frage 13: OUTCOMEINDIKATOREN bilden den Erfolg im Erreichen vordefinierter Behandlungsziele ab. Welche Art von Transparenz für externe Personen halten Sie für die Gruppe der Outcomeindikatoren für akzeptabel? "Meine Outcomeindikatoren zu teilen finde**  **ich akzeptabel mit: ..."** | | | | | | |
| --- | --- | --- | --- | --- | --- | --- |
|  | völlig inakzeptabel | eher inakzeptabel | neutral | eher akzeptabel | voll und ganz akzeptabel | kann ich nicht beantworten |
| a) Ärzten und Ärztinnen in meiner Gruppenpraxis |  |  |  |  |  |  |
| b) meinem Ärztenetzwerk |  |  |  |  |  |  |
| c) allen Ärzten und Ärztinnen meines Dachverbandes |  |  |  |  |  |  |
| d) den Krankenkassen |  |  |  |  |  |  |
| e) der Kantons- oder Bundesregierung |  |  |  |  |  |  |
| f) den Patient:innen der Praxis |  |  |  |  |  |  |
| g) der uneingeschränkten Allgemeinheit (z.B. frei zugängliche Daten im Internet) |  |  |  |  |  |  |

**Frage 14: Für wie akzeptabel halten Sie die Beteiligung folgender Instanzen am Prozess des Qualitätsmanagements für die Aufgaben der DATENERHEBUNG, -VERWALTUNG, -AUSWERTUNG und VERÖFFENTLICHUNG?**

völlig

inakzeptabel

eher

inakzeptabel

neutral eher

akzeptabel

voll und ganz

akzeptabel

kann ich nicht

beantworten

1. Eigene (Gruppen-)Praxis
2. Ärztenetzwerke
3. Fachgesellschaften
4. Krankenkassen
5. Kantons- oder fB)uAnkdaedseremgiisecrhuengInstitute
6. Private Anbieter
7. Betreiber meines Praxisinformationssystems

**Frage 15: Qualitätsdaten können auf unterschiedliche Wege erhoben werden. Welche METHODE der DATENERHEBUNG empfinden Sie als akzeptabel?**

völlig

inakzeptabel

eher

inakzeptabel

neutral eher

akzeptabel

voll und ganz

akzeptabel

kann ich nicht

beantworten

1. Selbstdeklaration
2. Erhebung durch Praxismitarbeiter
3. Erhebung durch dÄ)rzEtrehneebtzuwngerdkurch Zertifizierungsstelle (z.B. EQUAM)
4. Automatische Erhebung

anonymisierter Daten aus der elektronischen Krankengeschichte

1. Patientenbefragungssysteme (PROMS)

| **Frage 16: Welche Modelle von ANREIZEN oder SANKTIONEN im Rahmen von**  **Qualitätserhebungen empfinden Sie als akzeptabel?** | | | | | | |
| --- | --- | --- | --- | --- | --- | --- |
|  | völlig  inakzeptabel | eher  inakzeptabel | neutral | eher  akzeptabel | voll und ganz  akzeptabel | kann ich nicht  beantworten |

1. Sanktionen bei Verweigerung der Teilnahme
2. Bonus bei Teilnahme
3. Sanktionen bei Nichterreichen von Qualitätszielen
4. Bonus bei Erreichen von Qualitätszielen

Frage 17: Wie viel Ihrer ZEIT wären Sie persönlich 0h

bereit, auf MONATLICHER Basis in die Erhebung von < 1h

Qualitätsdaten zu investieren? 1-2h

bis zu einem halben Arbeitstag bis zu einem ganzen Arbeitstag

Demographie

Frage 18: Bitte wählen Sie Ihr Alter an: < 30 Jahre 30-39 Jahre

40-49 Jahre

50-59 Jahre

>60 Jahre

Frage 19: Bitte wählen Sie Ihr Geschlecht an: Weiblich Männlich Anderes keine Angabe

Frage 20: Welchen Facharzttitel tragen Sie Allgemeine Innere Medizin

(Mehrfachnennungen möglich)? Praktischer Arzt/Praktische Ärztin In Weiterbildung AIM

Andere

Frage 21: Wie viele Jahre Erfahrung in der Bis 10 Jahre

Hausarztmedizin haben Sie? 10-20 Jahre

Mehr als 20 Jahre

Frage 22: Führen Sie eine elektronische Krankenakte? Ja Nein

**Offene Kommentare**

Möchten Sie Ihren Antworten noch etwas hinzufügen?

Auf der nächsten Seite können Sie an der Verlosung

teilnehmen und die Umfrage abschliessen.

Ein herzliches Dankeschön an alle Teilnehmer:Innen dieser Befragung. Ihre Beiträge sind von unschätzbarem Wert und werden die Zukunft der Gesundheitsversorgung in der Schweiz positiv beeinflussen. Ihre Zeit und Ihr Engagement sind hochgeschätzt.

Um an der Verlosung der versprochenen Gutscheine im Wert von Fr. 300.- teilzunehmen, geben Sie bitte hier Ihre E-Mail-Adresse an.

Ihre E-Mail-Adresse: {mail}

Um die Umfrage abzuschliessen, drücken Sie bitte noch auf den Button "Übermitteln". Vielen herzlichen Dank!

David Wirth dipl. med.

Institut für Hausarztmedizin Universitätsspital Zürich Pestalozzistr. 24

CH-8091 Zürich

E-Mail: [david.wirth@usz.ch](mailto:david.wirth@usz.ch)
