# Supplementary material for: Acceptability of Components for a Mandatory Quality Improvement Framework: A Survey Among Swiss General Practitioners
Source: Health Serv Insights. 2025 Jun 21;18:11786329251346828. doi: 10.1177/11786329251346828 (PMC12182608; doi:10.1177/11786329251346828)
Supplement: sj-docx-2-his-10.1177_11786329251346828 – Supplemental material for Acceptability of Components for a Mandatory Quality Improvement Framework: A Survey Among Swiss General Practitioners [file sj-docx-2-his-10.1177_11786329251346828.docx]

## Supplementary Material: Tables and Figures

#### Supplementary table 1: Acceptance of different types of QIs

|  | level | self-employed | employed | p |
| --- | --- | --- | --- | --- |
| n |  | 121 | 102 |  |
| structural QIs: medical infrastructure (e.g. on site practice laboratory, x-ray, pharmacy) (%) | acceptable | 93 (79.5) | 83 (88.3) | 0.128 |
|  | neutral or unacceptable | 24 (20.5) | 11 (11.7) |  |
| structural QIs: personnel (e.g. experience years and qualification) (%) | acceptable | 80 (68.4) | 74 (78.7) | 0.127 |
|  | neutral or unacceptable | 37 (31.6) | 20 (21.3) |  |
| structural QIs: infrastructure for patients (e.g. number of parking slots, accessibility) (%) | acceptable | 75 (64.1) | 64 (68.1) | 0.645 |
|  | neutral or unacceptable | 42 (35.9) | 30 (31.9) |  |
| structural QIs: quality management (e.g. certificates, participation in quality circles) (%) | acceptable | 90 (76.9) | 80 (85.1) | 0.187 |
|  | neutral or unacceptable | 27 (23.1) | 14 (14.9) |  |
| process QIs: monitoring processes (e.g. blood pressure monitoring or smoking status monitoring in at risk populations) (%) | acceptable | 66 (56.9) | 58 (64.4) | 0.34 |
|  | neutral or unacceptable | 50 (43.1) | 32 (35.6) |  |
| process QIs: therapeutic processes (e.g. influenza vaccination or lipid lowering in at-risk populations) (%) | acceptable | 64 (55.2) | 56 (62.2) | 0.381 |
|  | neutral or unacceptable | 52 (44.8) | 34 (37.8) |  |
| outcome QIs: surrogate outcomes (e.g. hypertension control) (%) | acceptable | 51 (44.3) | 45 (50.6) | 0.459 |
|  | neutral or unacceptable | 64 (55.7) | 44 (49.4) |  |
| outcome QIs: hard outcomes (e.g. hospitalizations) (%) | acceptable | 49 (42.6) | 44 (49.4) | 0.407 |
|  | neutral or unacceptable | 66 (57.4) | 45 (50.6) |  |
| outcome QIs: patient reported experience measures (PREMS) (%) | acceptable | 46 (40.0) | 35 (39.3) | 1 |
|  | neutral or unacceptable | 69 (60.0) | 54 (60.7) |  |

#### Supplementary table 2: Acceptance of sharing achievement data from different types of QIs with specific bodies

|  | level | self-employed | employed | p |
| --- | --- | --- | --- | --- |
| n |  | 121 | 102 |  |
| Sharing achievement data of… |  | | | |
| …structural indicators with group practice (%) | acceptable | 105 (89.7) | 85 (93.4) | 0.494 |
|  | neutral or unacceptable | 12 (10.3) | 6 (6.6) |  |
| …structural indicators with physician network (%) | acceptable | 102 (87.2) | 82 (90.1) | 0.662 |
|  | neutral or unacceptable | 15 (12.8) | 9 (9.9) |  |
| …structural indicators with medical association (%) | acceptable | 71 (60.7) | 61 (67.0) | 0.425 |
|  | neutral or unacceptable | 46 (39.3) | 30 (33.0) |  |
| …structural indicators with healthcare insurance companies (%) | acceptable | 27 (23.1) | 22 (24.2) | 0.984 |
|  | neutral or unacceptable | 90 (76.9) | 69 (75.8) |  |
| …structural indicators with government authorities (%) | acceptable | 24 (20.5) | 29 (31.9) | 0.088 |
|  | neutral or unacceptable | 93 (79.5) | 62 (68.1) |  |
| …structural indicators with patients of the practice (%) | acceptable | 45 (38.5) | 50 (54.9) | 0.026 |
|  | neutral or unacceptable | 72 (61.5) | 41 (45.1) |  |
| …structural indicators with the general public (%) | acceptable | 13 (11.1) | 20 (22.0) | 0.053 |
|  | neutral or unacceptable | 104 (88.9) | 71 (78.0) |  |
| …process indicators with group practice (%) | acceptable | 103 (89.6) | 85 (95.5) | 0.193 |
|  | neutral or unacceptable | 12 (10.4) | 4 (4.5) |  |
| …process indicators with physician network (%) | acceptable | 98 (85.2) | 77 (86.5) | 0.951 |
|  | neutral or unacceptable | 17 (14.8) | 12 (13.5) |  |
| …process indicators with medical association (%) | acceptable | 56 (48.7) | 54 (60.7) | 0.119 |
|  | neutral or unacceptable | 59 (51.3) | 35 (39.3) |  |
| …process indicators with healthcare insurance companies (%) | acceptable | 8 (7.0) | 24 (27.0) | <0.001 |
|  | neutral or unacceptable | 107 (93.0) | 65 (73.0) |  |
| …process indicators with government authorities (%) | acceptable | 5 (4.3) | 19 (21.3) | <0.001 |
|  | neutral or unacceptable | 110 (95.7) | 70 (78.7) |  |
| …process indicators with patients of the practice (%) | acceptable | 25 (21.7) | 26 (29.2) | 0.289 |
|  | neutral or unacceptable | 90 (78.3) | 63 (70.8) |  |
| …process indicators with the general public (%) | acceptable | 3 (2.6) | 9 (10.1) | 0.05 |
|  | neutral or unacceptable | 112 (97.4) | 80 (89.9) |  |
| …outcome indicators with group practice (%) | acceptable | 97 (84.3) | 84 (95.5) | 0.022 |
|  | neutral or unacceptable | 18 (15.7) | 4 (4.5) |  |
| …outcome indicators with physician network (%) | acceptable | 91 (79.1) | 76 (86.4) | 0.249 |
|  | neutral or unacceptable | 24 (20.9) | 12 (13.6) |  |
| …outcome indicators with medical association (%) | acceptable | 47 (40.9) | 48 (54.5) | 0.073 |
|  | neutral or unacceptable | 68 (59.1) | 40 (45.5) |  |
| …outcome indicators with healthcare insurance companies (%) | acceptable | 8 (7.0) | 17 (19.3) | 0.015 |
|  | neutral or unacceptable | 107 (93.0) | 71 (80.7) |  |
| …outcome indicators with government authorities (%) | acceptable | 7 (6.1) | 16 (18.2) | 0.013 |
|  | neutral or unacceptable | 108 (93.9) | 72 (81.8) |  |
| …outcome indicators with patients of the practice (%) | acceptable | 20 (17.4) | 24 (27.3) | 0.128 |
|  | neutral or unacceptable | 95 (82.6) | 64 (72.7) |  |
| …outcome indicators with the general public (%) | acceptable | 4 (3.5) | 8 (9.1) | 0.168 |
|  | neutral or unacceptable | 111 (96.5) | 80 (90.9) |  |

#### Supplementary table 3: Acceptance of entities establishing QIs, gathering and managing QI data

|  | Level | self-employed | employed | p |
| --- | --- | --- | --- | --- |
| n |  | 121 | 102 |  |
| QIs established by physician networks (%) | acceptable | 108 (89.3) | 90 (88.2) | 0.978 |
|  | neutral or unacceptable | 13 (10.7) | 12 (11.8) |  |
| QIs established by medical associations (%) | acceptable | 93 (76.9) | 89 (87.3) | 0.068 |
|  | neutral or unacceptable | 28 (23.1) | 13 (12.7) |  |
| QIs established by government authorities (%) | acceptable | 8 (6.6) | 21 (20.6) | 0.004 |
|  | neutral or unacceptable | 113 (93.4) | 81 (79.4) |  |
| QIs established by healthcare insurance companies (%) | acceptable | 4 (3.3) | 17 (16.7) | 0.002 |
|  | neutral or unacceptable | 117 (96.7) | 85 (83.3) |  |
| QIs established by certification agencies (%) | acceptable | 44 (36.4) | 56 (54.9) | 0.008 |
|  | neutral or unacceptable | 77 (63.6) | 46 (45.1) |  |
| QIs established by academic institution (%) | acceptable | 94 (77.7) | 88 (86.3) | 0.14 |
|  | neutral or unacceptable | 27 (22.3) | 14 (13.7) |  |
| QIs established by patient organizations (%) | acceptable | 29 (24.0) | 38 (37.3) | 0.044 |
|  | neutral or unacceptable | 92 (76.0) | 64 (62.7) |  |
| QIs established by international organizations (%) | acceptable | 9 (7.4) | 12 (11.8) | 0.383 |
|  | neutral or unacceptable | 112 (92.6) | 90 (88.2) |  |
| data gathering by: self declaration (%) | acceptable | 78 (67.8) | 65 (73.9) | 0.436 |
|  | neutral or unacceptable | 37 (32.2) | 23 (26.1) |  |
| data gathering by: practice staff (%) | acceptable | 78 (67.8) | 69 (78.4) | 0.13 |
|  | neutral or unacceptable | 37 (32.2) | 19 (21.6) |  |
| data gathering by: physician networks (%) | acceptable | 80 (69.6) | 57 (64.8) | 0.568 |
|  | neutral or unacceptable | 35 (30.4) | 31 (35.2) |  |
| data gathering by: certification agency (e.g. EQUAM) (%) | acceptable | 60 (52.2) | 59 (67.0) | 0.047 |
|  | neutral or unacceptable | 55 (47.8) | 29 (33.0) |  |
| data gathering by: routine data from electronic medical records (%) | acceptable | 70 (60.9) | 50 (56.8) | 0.662 |
|  | neutral or unacceptable | 45 (39.1) | 38 (43.2) |  |
| data gathering by: patient experiences (%) | acceptable | 40 (34.8) | 39 (44.3) | 0.217 |
|  | neutral or unacceptable | 75 (65.2) | 49 (55.7) |  |
| data management by: group practice (%) | acceptable | 96 (83.5) | 75 (85.2) | 0.885 |
|  | neutral or unacceptable | 19 (16.5) | 13 (14.8) |  |
| data management by: physician networks (%) | acceptable | 98 (85.2) | 69 (78.4) | 0.283 |
|  | neutral or unacceptable | 17 (14.8) | 19 (21.6) |  |
| data management by: medical associations (%) | acceptable | 67 (58.3) | 62 (70.5) | 0.101 |
|  | neutral or unacceptable | 48 (41.7) | 26 (29.5) |  |
| data management by: healthcare insurance companies (%) | acceptable | 3 (2.6) | 16 (18.2) | <0.001 |
|  | neutral or unacceptable | 112 (97.4) | 72 (81.8) |  |
| data management by: government authorities (%) | acceptable | 3 (2.6) | 17 (19.3) | <0.001 |
|  | neutral or unacceptable | 112 (97.4) | 71 (80.7) |  |
| data management by: academic institutions (%) | acceptable | 66 (57.4) | 60 (68.2) | 0.154 |
|  | neutral or unacceptable | 49 (42.6) | 28 (31.8) |  |
| data management by: private companies (%) | acceptable | 6 (5.2) | 7 (8.0) | 0.617 |
|  | neutral or unacceptable | 109 (94.8) | 81 (92.0) |  |
| data management by: practice information system developers (%) | acceptable | 32 (27.8) | 23 (26.1) | 0.913 |
|  | neutral or unacceptable | 83 (72.2) | 65 (73.9) |  |

#### Supplementary table 4: Acceptance of financing sources and incentives

|  | level | self-employed | employed | p |
| --- | --- | --- | --- | --- |
| n |  | 121 | 102 |  |
| financing by group practice (%) | acceptable | 8 (6.7) | 7 (7.1) | 1 |
|  | neutral or unacceptable | 112 (93.3) | 92 (92.9) |  |
| financing by physician network (%) | acceptable | 38 (31.7) | 33 (33.3) | 0.907 |
|  | neutral or unacceptable | 82 (68.3) | 66 (66.7) |  |
| financing by healthcare insurance companies (%) | acceptable | 48 (40.0) | 39 (39.4) | 1 |
|  | neutral or unacceptable | 72 (60.0) | 60 (60.6) |  |
| financing by quality contracts (%) | acceptable | 69 (57.5) | 59 (59.6) | 0.861 |
|  | neutral or unacceptable | 51 (42.5) | 40 (40.4) |  |
| financing by medical tariff (%) | acceptable | 96 (80.0) | 80 (80.8) | 1 |
|  | neutral or unacceptable | 24 (20.0) | 19 (19.2) |  |
| financing by government authorities (%) | acceptable | 79 (65.8) | 74 (74.7) | 0.2 |
|  | neutral or unacceptable | 41 (34.2) | 25 (25.3) |  |
| sanction for non-participation (%) | acceptable | 15 (13.0) | 17 (19.5) | 0.29 |
|  | neutral or unacceptable | 100 (87.0) | 70 (80.5) |  |
| bonus for participation (%) | acceptable | 87 (75.7) | 71 (81.6) | 0.399 |
|  | neutral or unacceptable | 28 (24.3) | 16 (18.4) |  |
| sanction for non-achievement (%) | acceptable | 8 (7.0) | 9 (10.3) | 0.546 |
|  | neutral or unacceptable | 107 (93.0) | 78 (89.7) |  |
| bonus for achievement (%) | acceptable | 78 (67.8) | 66 (75.9) | 0.274 |
|  | neutral or unacceptable | 37 (32.2) | 21 (24.1) |  |

#### Supplementary table 5: Model output for employed vs. self-employed (reference) GPs’ likelihood to accept components of framework conditions for mandatory quality improvement adjusted for GP sex, years of practice experience, and previous participation in quality improvement initiatives (coefficients not shown). Adjustment of p value was performed by multiplication by the number of outcomes evaluated (that is n=62).

| Component of framework for mandatory quality improvement | odds ratio | confidence interval | adjusted p | unadjusted p |
| --- | --- | --- | --- | --- |
| sharing achievement of process QIs with healthcare insurances companies | 5.9 | 2.5 to 15.3 | 0.008 | <0.001 |
| sharing achievement of process QIs with government authorities | 7.4 | 2.7 to 24.4 | 0.018 | <0.001 |
| involvement in QI data management: government authorities | 9.9 | 3 to 45.3 | 0.038 | 0.001 |
| QIs established by healthcare insurance companies | 7.8 | 2.6 to 29.8 | 0.048 | 0.001 |
| involvement in QI data management: healthcare insurance companies | 7.9 | 2.4 to 35.8 | 0.123 | 0.002 |
| QIs established by government authorities | 4.4 | 1.8 to 12.4 | 0.155 | 0.003 |
| sharing achievement of outcome QIs with healthcare insurance companies | 4.3 | 1.7 to 11.6 | 0.183 | 0.003 |
| sharing achievement of outcome QIs with government authorities | 3.6 | 1.4 to 10.5 | 0.703 | 0.011 |
| sharing achievement of outcome QIs with group practice | 4.7 | 1.5 to 18 | 0.709 | 0.011 |
| sharing achievement of process QIs with the general public | 6.0 | 1.6 to 29.4 | 0.759 | 0.012 |
| QIs established by certification agencies | 2.1 | 1.2 to 3.9 | 0.818 | 0.013 |
| QIs established by patient organizations | 2.2 | 1.2 to 4.3 | 0.860 | 0.014 |
| sharing structural QIs with the general public | 2.6 | 1.2 to 6 | 1.000 | 0.022 |
| sharing achievement of outcome QIs with medical association | 2.0 | 1.1 to 3.7 | 1.000 | 0.022 |
| sharing achievement of outcome QIs with the general public | 4.4 | 1.2 to 18.5 | 1.000 | 0.027 |
| sharing achievement of structural QIs with patients of the practice | 1.9 | 1.1 to 3.6 | 1.000 | 0.028 |
| involvement in QI data gathering: certification agency | 2.0 | 1.1 to 3.6 | 1.000 | 0.031 |
| sharing achievement of outcome QIs with patients of the practice | 2.2 | 1.1 to 4.6 | 1.000 | 0.035 |
| QI measurement financing by government authorities | 2.0 | 1 to 4 | 1.000 | 0.040 |
| involvement in QI data gathering: practice staff | 2.0 | 1 to 4 | 1.000 | 0.045 |
| sharing achievement of process QIs with medical association | 1.8 | 1 to 3.4 | 1.000 | 0.047 |
| sharing achievement of process QIs with group practice | 3.0 | 0.9 to 11.9 | 1.000 | 0.093 |
| sanctions for non-participation | 2.0 | 0.9 to 4.7 | 1.000 | 0.094 |
| sharing achievement of structural QIs with government authorities | 1.8 | 0.9 to 3.5 | 1.000 | 0.094 |
| sharing achievement of process QIs with patients of the practice | 1.8 | 0.9 to 3.5 | 1.000 | 0.107 |
| involvement in QI data management: medical associations | 1.7 | 0.9 to 3.2 | 1.000 | 0.112 |
| involvement in QI data gathering: patient experiences | 1.6 | 0.9 to 2.9 | 1.000 | 0.146 |
| sharing achievement of outcome QIs with physician network | 1.8 | 0.8 to 4.1 | 1.000 | 0.146 |
| QIs established by academic institution | 1.8 | 0.8 to 4.3 | 1.000 | 0.167 |
| QIs established by medical associations | 1.8 | 0.8 to 4.1 | 1.000 | 0.169 |
| involvement in QI data management: academic institutions | 1.5 | 0.8 to 2.7 | 1.000 | 0.219 |
| structural QIs pertaining to personell | 1.5 | 0.8 to 3 | 1.000 | 0.224 |
| involvement in QI data management: group practice | 1.7 | 0.7 to 3.9 | 1.000 | 0.230 |
| structural QIs pertaining to quality management | 1.6 | 0.7 to 3.5 | 1.000 | 0.243 |
| involvement in QI data gathering: self declaration | 1.5 | 0.8 to 2.8 | 1.000 | 0.262 |
| involvement in QI data management: private companies | 1.9 | 0.6 to 6.6 | 1.000 | 0.283 |
| sharing achievement of structural QIs with group practice | 1.9 | 0.6 to 6.8 | 1.000 | 0.283 |
| structural QIs pertaining to medical infrastructure | 1.5 | 0.7 to 3.6 | 1.000 | 0.308 |
| outcome QIs: hard outcomes | 1.3 | 0.7 to 2.4 | 1.000 | 0.321 |
| outcome QIs: surrogate outcomes | 1.3 | 0.7 to 2.4 | 1.000 | 0.335 |
| bonus for achievement | 1.4 | 0.7 to 2.7 | 1.000 | 0.346 |
| bonuses for participation | 1.4 | 0.7 to 3 | 1.000 | 0.358 |
| sharing achievement of structural QIs with medical association | 1.3 | 0.7 to 2.5 | 1.000 | 0.392 |
| process QIs: clinical monitoring processes | 1.3 | 0.7 to 2.4 | 1.000 | 0.406 |
| QI measurement financing by healthcare insurance companies | 1.3 | 0.7 to 2.4 | 1.000 | 0.411 |
| QIs established by physician networks | 0.7 | 0.3 to 1.8 | 1.000 | 0.450 |
| process QIs: therapeutic processes | 1.2 | 0.7 to 2.3 | 1.000 | 0.482 |
| QIs established by international organizations | 1.4 | 0.5 to 3.8 | 1.000 | 0.486 |
| involvement in QI data management: physician networks | 0.8 | 0.4 to 1.7 | 1.000 | 0.548 |
| sharing achievement of structural QIs with physician network | 1.3 | 0.5 to 3.5 | 1.000 | 0.594 |
| sanctions for non-achievement | 1.3 | 0.5 to 3.9 | 1.000 | 0.604 |
| QI measurement financing by quality contracts | 1.2 | 0.6 to 2.1 | 1.000 | 0.615 |
| QI measurement financing by medical tariff | 1.2 | 0.6 to 2.6 | 1.000 | 0.616 |
| sharing achievement of process QIs with physician network | 1.2 | 0.5 to 2.9 | 1.000 | 0.619 |
| QI measurement financing by group practice | 1.3 | 0.4 to 4.5 | 1.000 | 0.634 |
| QI measurement financing by physician network | 1.2 | 0.6 to 2.2 | 1.000 | 0.636 |
| involvement in QI data gathering: routine data from electronic medical records | 0.9 | 0.5 to 1.7 | 1.000 | 0.754 |
| involvement in QI data gathering: physician networks | 0.9 | 0.5 to 1.7 | 1.000 | 0.782 |
| involvement in QI data management: practice information system developers | 0.9 | 0.5 to 1.8 | 1.000 | 0.839 |
| structural Qis pertaining to infrastructure for patients | 1.0 | 0.6 to 1.9 | 1.000 | 0.918 |
| outcome Qis: patient reported experience measures (PREMS) | 1.0 | 0.5 to 1.8 | 1.000 | 0.930 |
| sharing achievement of structural QIs with healthcare insurance companies | 1.0 | 0.5 to 2 | 1.000 | 0.979 |

#### Supplementary Figure 1: Multivariate logistic regression model of employed vs. self-employed GPs’ acceptance of framework conditions for quality improvement adjusted for working experience, sex and previous participation in quality improvement initiatives.


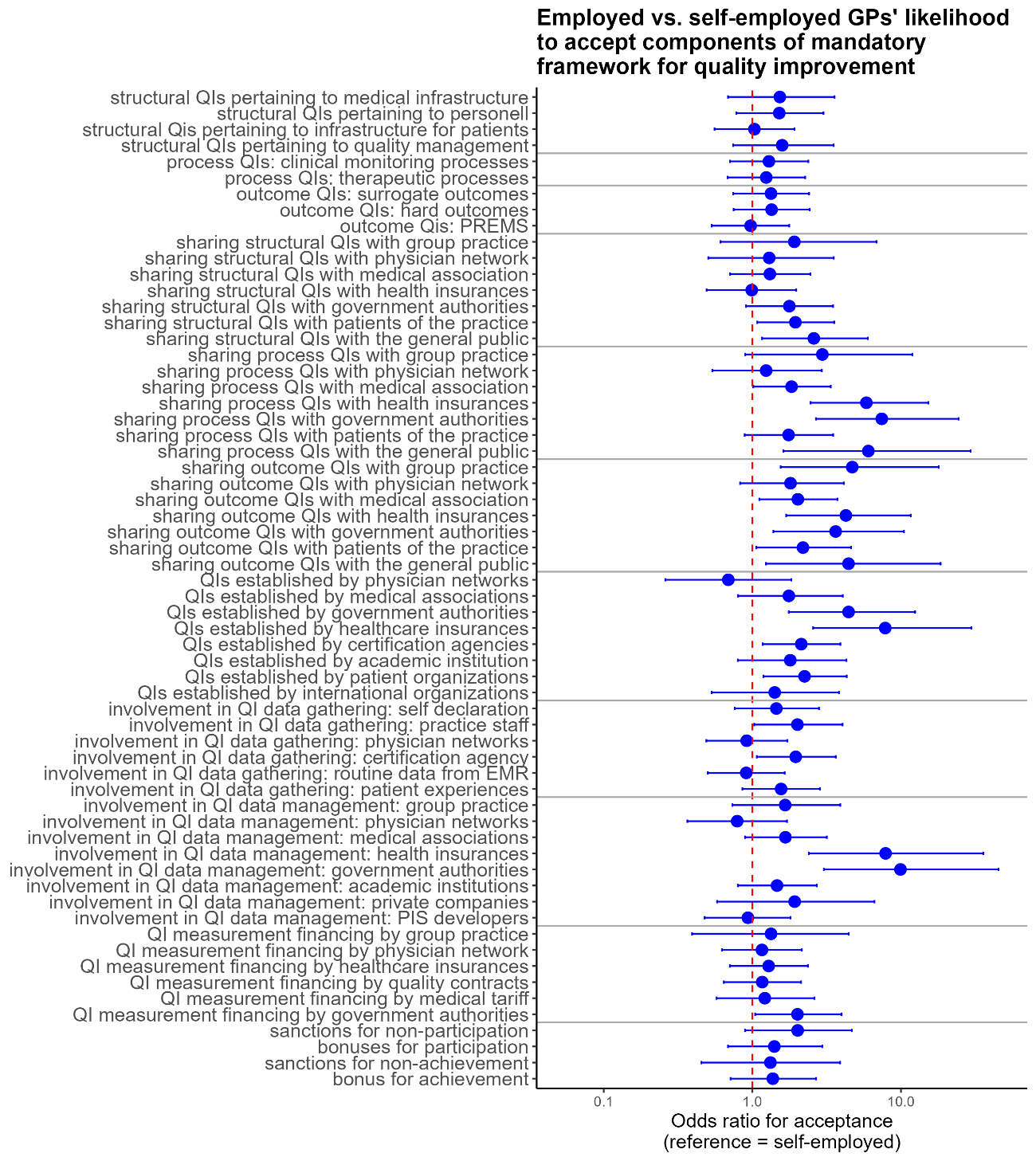


Figure Legend: Multivariate Logistic regression model results showing odds ratios (points) and 95% confidence intervals (whiskers) of employed GPs (reference self-employed) to rate specific components of a framework for mandatory quality improvement (y-axis) as acceptable vs. neutral or unacceptable. The model is adjusted for GP working experience, sex and previous participation in quality improvement initiatives.

Abbreviations: PREMS: Patient reported experience measures; EMR: Electronic medical records; PIS: Practice information system.
